# Characteristics, Treatment Patterns, Healthcare Resource Utilization, and Costs Among Patients with Multifocal Motor Neuropathy: A US Claims Database Cohort Study

**DOI:** 10.36469/001c.140817

**Published:** 2025-06-26

**Authors:** Nikhil Khandelwal, Marie Sanchirico, Ade Ajibade, Kiraat Munshi, Michelle Vu, Nicole Engel-Nitz, Christina Steiger, Amy J Anderson, Chafic Karam

**Affiliations:** 1 Takeda Pharmaceuticals USA, Lexington, Massachusetts; 2 Takeda Pharmaceuticals USA, Inc., Lexington, Massachusetts; 3 Optum, Inc., Eden Prairie, Minnesota, USA; 4 University of Pennsylvania Health System, Philadelphia, Pennsylvania, USA

**Keywords:** healthcare resource utilization, multifocal motor neuropathy, differential diagnoses, intravenous immunoglobulin, US claims database

## Abstract

**Background:** Multifocal motor neuropathy (MMN) is a rare, slowly progressive nerve disorder characterized by asymmetric limb weakness without sensory abnormalities. MMN is often misdiagnosed due to similarities in clinical symptoms with conditions including amyotrophic lateral sclerosis (ALS), making diagnosis and treatment challenging.

**Objectives:** This study assessed patient characteristics, treatment patterns, and the economic burden of MMN in the United States.

**Methods:** Using the Optum Research Database, this retrospective analysis included patients with ≥1 diagnostic or nondiagnostic medical claim with an MMN diagnosis code between 2016 and 2020 (date of first diagnosis-related claim =index date), and continuous enrollment for 12 months preindex and postindex. Patients with MMN within this group were identified using more specific criteria; ≥2 MMN nondiagnostic claims separated by ≥30 days, with no subsequent ALS diagnosis during follow-up. All patients who did not meet these criteria were included in the MMN-mimic cohort. Outcomes included treatment patterns, differential diagnoses, healthcare utilization, and costs.

**Results:** Of 904 patients identified, 37% had MMN and 63% had an MMN-mimic condition. Patients with MMN were significantly younger than patients in the MMN-mimic cohort (mean, 64.9 vs 66.8 years; *P* = .047). At preindex, significantly more patients with MMN received MMN-related medications than patients in the MMN-mimic cohort (20.5% vs 9.0%, respectively; *P* < .001). Intravenous immunoglobulin (IVIG) was the most common MMN-related medication. At postindex, more patients with MMN used IVIG (28.0%) compared with preindex (16.4%). In the 12 months preindex and postindex, >70% of patients had ≥1 differential diagnosis. The MMN cohort had higher all-cause total costs than the MMN-mimic cohort (mean preindex, 58 974vs48 132, respectively [*P* = .100]; mean postindex, 74 187vs50 652 [P = .002]); they also had significantly higher MMN-related healthcare costs (mean preindex, 23 625vs12 890 [*P* = .011]; mean postindex, 39 521vs11 938 [*P* < .001]).

**Discussion:** This study showed that most patients with initial MMN diagnoses had an alternative disorder after subsequent evaluation/follow-up, and patients with MMN incurred higher costs. Many patients with MMN did not receive IVIG, suggesting that undertreatment or misattribution of diagnosis codes are common.

**Conclusions:** Further education is needed regarding accurate diagnosis of MMN to ensure patient access to guideline-recommended treatment.

## INTRODUCTION

Multifocal motor neuropathy (MMN) is a rare nerve disorder in which the core clinical criteria for diagnosis include slowly progressive, focal, asymmetric limb weakness in the absence of sensory abnormalities.^1,2^ The disease is more common in men and is typically diagnosed in individuals between 30 and 50 years of age.^1,3^ Currently, the worldwide prevalence of MMN is estimated to be less than 1 in 100 000 people.^1^

MMN is often misdiagnosed and underdiagnosed because the clinical symptoms of the disease mimic other conditions including amyotrophic lateral sclerosis (ALS) and chronic inflammatory demyelinating polyradiculoneuropathy (CIDP).^2,4,5^ The similarities across these disorders make MMN difficult to diagnose in a timely manner, and delay the initiation of proper treatment. Intravenous immunoglobulin (IVIG) is the only US Food and Drug Administration-approved treatment option for patients with MMN.^6^ Accurate diagnosis of MMN can decrease the risk of inappropriate exposure to therapies used to treat other disorders, and it provides a better prognosis. While MMN is treatable and associated with a normal life expectancy, patients with ALS have a median survival of 3 to 5 years. Thus, correct diagnoses of MMN may allow patients to avoid the psychological effects of being misdiagnosed.^4^

There is limited evidence on the characteristics, treatment patterns, and economic burden of MMN. A potential factor responsible for the paucity of evidence is the dispersed centers of excellence for MMN, and the decentralized or non-single-payer system in the United States (compared with European and other countries) that leads to patients being treated at separate clinics across the country and not in a single center.^7^ The scattered nature of the healthcare system caring for these patients may lead to varying approaches to diagnosis and treatment, and can make the centralized and consistent collection of data on a patient’s healthcare journey cumbersome.

To address this evidence gap, this study used administrative claims data to assess characteristics, treatment patterns, and associated healthcare burden (ie, resource use and costs) among patients with MMN and compared them with those with MMN-mimic conditions.

## METHODS

This retrospective, observational study evaluated patients covered by commercial and Medicare Advantage plans using administrative claims from the Optum Research Database (Oct 1, 2015–Dec. 31, 2021). The Optum Research Database, Optum’s Health Insurance Portability and Accountability Act (HIPAA)–compliant proprietary administrative claims research database, comprises medical and pharmacy claims data (including linked enrollment) from 1993 to the present for more than 73 million patients in the United States.

**Figure 1.**
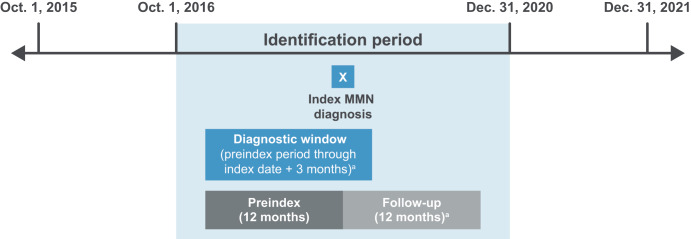
Study Design Flowchart Abbreviation: MMN, multifocal motor neuropathy. ^a^Note: Window end date was less if death was observed.

The study design is shown in **Figure 1**. The index date was defined as the date of the first medical claim (including claims for diagnostic [eg, laboratory or radiology tests] or nondiagnostic services [eg, treatment and management of conditions]), associated with a diagnosis of MMN (*International Classification of Diseases, Tenth Revision, Clinical Modification* [ICD-10-CM]: G61.82) during the identification period (Oct. 1, 2016–Dec. 31, 2020); the preindex period was defined as the 12-month period before the index date; the postindex follow-up period started on the index date and lasted for 12 months (or less if death was observed); the diagnostic window was defined as the period from the start of the preindex period through the first 3 months of the follow-up period (or less if death was observed), and was used to assess MMN-related diagnostic tests and evaluations surrounding the index date.

All patients included in the study had at least 1 diagnostic or nondiagnostic claim during the identification period associated with a diagnosis of MMN using the ICD-10-CM and were continuously enrolled in medical and pharmacy benefits for 12 months before and after the index date. Patients were further stratified into two cohorts: an MMN cohort and an MMN-mimic cohort. Patients in the MMN cohort were identified based on having at least 2 MMN nondiagnostic medical claims separated by at least 30 days, with no subsequent ALS diagnosis reported during the postindex follow-up period. The definition for the MMN cohort was developed based on the European Federation of Neurological Sciences/Peripheral Nerve Society diagnostic guidelines and the American Association of Neuromuscular and Electrodiagnostic Medicine consensus criteria,^2,8^ with reference to more recent insights on MMN and MMN-like conditions,^9^ and following review of ICD-10-CM code data by a neuromuscular expert (C.K.). The MMN-mimic cohort included all patients who satisfied the overall study inclusion criteria who were not selected for inclusion in the MMN cohort (see **Supplementary Table S1** for a list of ICD-10-CM codes related to MMN-mimic conditions). Treatments were categorized according to whether they were relevant for MMN or for MMN-mimic conditions: MMN-related medications included IVIG, subcutaneous immunoglobulin (SCIG), and immunosuppressives; MMN-mimic-related treatments included systemic corticosteroids, medications for ALS, neuropathic pain, and opioid or non-opioid pain medications.

Results were reported for the preindex and postindex follow-up periods. Study outcomes assessed included treatment patterns, differential diagnoses considered for MMN and MMN-mimic conditions, all-cause healthcare resource utilization (HCRU) and costs (2021 US dollar [USD], COB [coordination of benefits]-adjusted), and MMN-related HCRU and costs (2021 USD, COB-adjusted). HCRU and costs were defined as MMN-related if claims were associated with an MMN diagnosis code or a code for a medication/diagnostic test for MMN. Further information regarding MMN-related diagnostic procedures and medications and MMN-mimic differential diagnoses and treatments are presented in the **Online Supplementary Material**.

All statistical analyses were performed using SAS v9.4 (SAS Institute) or later version.^10^ Numbers and percentages were provided for categorical variables. Means, medians, and standard deviations were provided for continuous variables. The conventional significance level ɑ = 0.05 on a 2-tailed test was used to determine statistical significance. This retrospective analysis of de-identified administrative claims is exempt from human subject’s ethical research requirements and did not require Institutional Review Board or Ethics Committee approval nor informed consent.

## RESULTS

### Patient Characteristics

Overall, 904 patients had at least 1 claim for MMN during the identification period and were also continuously enrolled during the 12-month preindex and postindex periods. Of these patients, 336 (37%) met the study definition of MMN and the remaining 568 (63%) patients were classified into the MMN-mimic cohort. Patients with MMN in this study were significantly younger than those with MMN-mimic conditions (mean, 64.9 vs 66.8 years, respectively; *P* = .047) (**Table 1**). Approximately two-thirds of patients were aged 65 years or older and were covered by Medicare. Patients in the MMN cohort also had longer follow-up periods than the MMN-mimic cohort (mean, 31.8 vs 27.2 months, respectively; *P* < .001). Proportions of patients with claims during the diagnostic window associated with MMN-related diagnostic procedures were generally similar for MMN and MMN-mimic cohorts, with the exception of spinal MRI, which was more commonly performed in the MMN group. Specialties of the physicians submitting claims related to diagnostic tests for MMN can be seen in **Supplementary Table S2**.

**Table 1.** Demographic Characteristics of the Study Population

**Characteristics**	**Overall Study Population (N = 904)**	**MMN (n = 336)**	**MMN-Mimic (n = 568)**	**MMN vs MMN-** **Mimic *P* Value**
Age at index date or most recent claim after index date, y				
Mean (SD)	66.1 (13.8)	64.9 (14.1)	66.8 (13.6)	0.047^a^
<18-44, n (%)	62 (6.9)	25 (7.4)	37 (6.5)	0.594
45-64, n (%)	304 (33.6)	128 (38.1)	176 (31.0)	0.029^a^
≥ 65, n (%)	538 (59.5)	183 (54.5)	355 (62.5)	0.017^a^
Sex, n (%)^b^				
Men	490 (54.2)	186 (55.4)	304 (53.5)	0.592
Women	413 (45.7)	150 (44.6)	263 (46.3)	0.628
Race, n (%)				
White	569 (62.9)	224 (66.7)	345 (60.7)	0.075
African American or Black	111 (12.3)	42 (12.5)	69 (12.2)	0.876
Hispanic	91 (10.1)	28 (8.3)	63 (11.1)	0.183
Asian	17 (1.9)	7 (2.1)	10 (1.8)	0.730
Unknown	116 (12.8)	35 (10.4)	81 (14.3)	0.095
Insurance type, n (%)^c^				
Medicare	609 (67.4)	220 (65.5)	389 (68.5)	0.351
Commercial	294 (32.5)	116 (34.5)	178 (31.3)	0.323
Region, n (%)				0.578
South	433 (47.9)	156 (46.4)	277 (48.8)	0.496
Midwest	206 (22.8)	79 (23.5)	127 (22.4)	0.690
Northeast	149 (16.5)	52 (15.5)	97 (17.1)	0.531
West	116 (12.8)	49 (14.6)	67 (11.8)	0.226
Baseline Charlson Comorbidity Index score, patient n (%)				
Mean (SD)	1.91 (2.2)	1.81 (2.2)	1.97 (2.3)	0.311
Categorical				0.666
0	324 (35.8)	129 (38.4)	195 (34.3)	0.218
1-2	316 (35.0)	114 (33.9)	202 (35.6)	0.618
3-4	146 (16.2)	51 (15.2)	95 (16.7)	0.541
5+	118 (13.1)	42 (12.5)	76 (13.4)	0.704
MMN-related diagnostic procedures, n (%)^d^				
Electrophysiological analysis	345 (38.2)	137 (40.8)	208 (36.6)	0.214
Spinal MRI	206 (22.8)	91 (27.1)	115 (20.3)	0.018
Lumbar puncture	64 (7.1)	27 (8.0)	37 (6.5)	0.389
MMN-related antibody tests	<11 (<1.2)	6 (1.8)	<5 (<0.9)	0.133

Abbreviations: MMN, multifocal motor neuropathy; MRI, magnetic resonance imaging.

^a^Statistically significant (*P* < .05) difference.

^b^Patients with missing sex data were identified; data are not disclosed owing to small sample sizes.

^c^Patients with missing insurance type data were identified; data are not disclosed owing to small sample sizes.

^d^Data are masked where patient numbers are <5.

### Treatment Patterns in MMN and MMN-Mimic Cohorts During the Preindex and Postindex Periods

During the preindex period, a significantly higher proportion of patients with MMN compared with the MMN-mimic cohort received MMN-related medications (20.5% vs 9.0%, respectively; *P* < .001) and corticosteroids (50.3% vs 43.3%, respectively; *P* = .042) (**Table 2**). IVIG was the most common MMN-related medication used across both cohorts in the preindex period (MMN, 16.4%; MMN-mimic, 4.9%) (**Table 2**). SCIG was not prescribed during the preindex period. A significantly higher proportion of patients in the MMN cohort received systemic corticosteroids compared with the MMN-mimic cohort (50.3% vs 43.3%, respectively; *P* = .042).

**Table 2.** Selected Treatment Patterns in the Study Population During the Preindex and Postindex Periods

**Characteristics**	**Overall Study Population (N = 904)**	**MMN (n = 336)**	**MMN-Mimic (n = 568)**	**MMN vs MMN-** **Mimic *P* Value**
Preindex period				
MMN-related disease-modifying medication use,^a^ n (%)	120 (13.3)	69 (20.5)	51 (9.0)	<.001^c^
IVIG, n (%)	83 (9.2)	55 (16.4)	28 (4.9)	<.001^c^
Immunosuppressives, n (%)	46 (5.1)	20 (6.0)	26 (4.6)	.363
MMN-mimic related medication use,^b^ n (%)	681 (75.3)	255 (75.9)	426 (75.0)	.763
Systemic corticosteroids, n (%)	415 (45.9)	169 (50.3)	246 (43.3)	.042^c^
Postindex period				
MMN-related disease-modifying medication use,^a^ n (%)	190 (21.0)	107 (31.9)	83 (14.6)	<.001^c^
IVIG, n (%)	155 (17.2)	94 (28.0)	61 (10.7)	<.001^c^
Immunosuppressives, n (%)	50 (5.5)	25 (7.4)	25 (4.4)	.053
MMN-mimic related medication use,^b^ n (%)	695 (76.9)	271 (80.7)	424 (74.7)	.038^c^
Systemic corticosteroids, n (%)	411 (45.5)	164 (48.8)	247 (43.5)	.120

Abbreviations: ALS, amyotrophic lateral sclerosis; IVIG, intravenous immunoglobulin; MMN, multifocal motor neuropathy.

^a^Other MMN-related treatments not shown in this table included immunologics/immunosuppressives.

^b^Other MMN-mimic related treatments not shown in this table included medications for ALS, neuropathic pain, and opioid or non-opioid pain medications.

^c^Statistically significant (*P* <.05) difference.

During the postindex period, the proportion of patients with MMN using IVIG was higher than it was during the preindex period (preindex, 16.4%; postindex, 28.0%) (**Table 2**). Fewer than 5 patients were prescribed SCIG during follow-up. A higher proportion of the MMN cohort was treated with immunosuppressive medication (7.4% vs 4.4%, respectively; *P* value nonsignificant) and systemic corticosteroids (48.8% vs 43.5%, respectively; *P* value nonsignificant) compared with the MMN-mimic cohort.

### MMN Differential Diagnoses During Preindex and Postindex Periods

**Figure 2.**
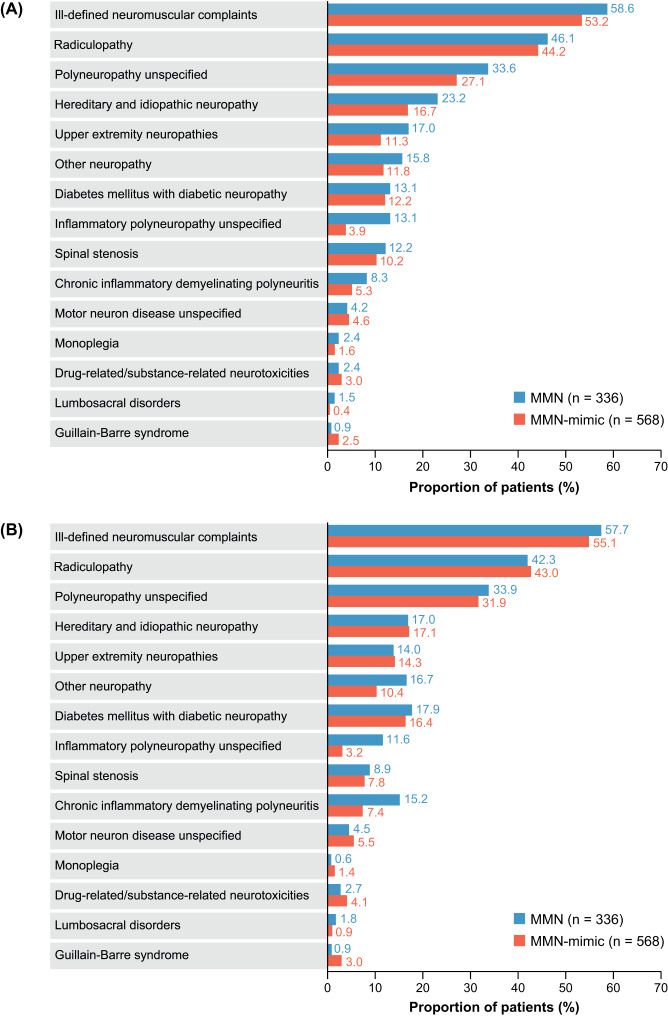
Selected^a^ Differential Diagnoses in the Study Population in the 12 Months (**A**) Preindex and (**B**) Postindex Abbreviation: MMN, multifocal motor neuropathy. ^a^Selected by frequency and conditions cited as common differential diagnoses.

For the 12 months preindex and postindex, more than 70% of patients in both cohorts had at least 1 differential diagnosis. During the preindex period, the most common diagnosis was for ill-defined neuromuscular complaints (MMN, 58.6%; MMN-mimic, 53.2%), followed by radiculopathy (MMN, 46.1%; MMN-mimic, 44.2%), polyneuropathy unspecified (MMN, 33.6%; MMN-mimic, 27.1%), and hereditary and idiopathic neuropathy (MMN, 23.2%; MMN-mimic, 16.7%) (**Figure 2A, B**). Of note, diabetes mellitus with diabetic neuropathy was also reported (MMN, 13.1%; MMN-mimic, 12.2%). The proportions of patients with diagnoses for CIDP were higher in both cohorts during the postindex period (MMN, 15.2%; MMN-mimic, 7.4%) compared with the preindex period (MMN: 8.3%; MMN-mimic: 5.3%).

### All-Cause HCRU and Costs in MMN and MMN-Mimic Cohorts During the Preindex and Postindex Periods

During the preindex period (**Supplementary Figure S1A**), the proportion of patients in the MMN cohort (vs MMN-mimic cohort) with inpatient stays was significantly lower (21% vs 31%, respectively; *P* = .001). Ambulatory visits, pharmacy use, and emergency room visits were comparable between cohorts.

**Figure 3.**
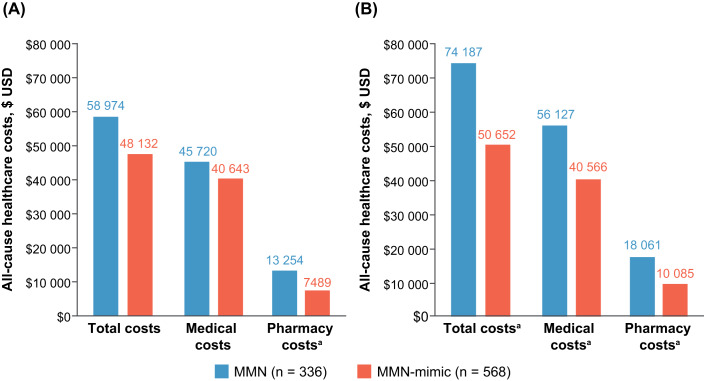
Mean All-Cause Costs (2021 USD) in the Study Population During the (**A**) Preindex and (**B**) Postindex Period Abbreviation: MMN, multifocal motor neuropathy; USD, US dollars. ^a^Statistically significant (*P* < .05) difference between MMN and MMN-mimic cohorts.

**Figure 4.**
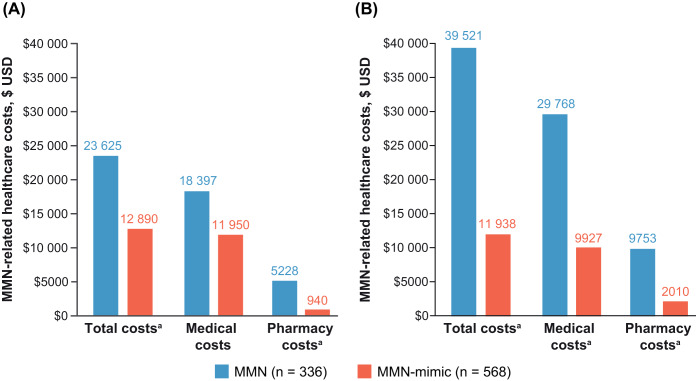
Mean MMN-Related Costs (2021 USD) in the Study Population During the (**A**) Preindex and (**B**) Postindex Period Abbreviations: MMN, multifocal motor neuropathy; USD, US dollars. Note: Costs were defined as MMN-related if claims had an MMN diagnosis code or had a code for a medication/diagnostic test for MMN. ^a^Statistically significant (*P* < .05) difference between MMN and MMN-mimic cohorts.

Throughout the postindex period, all-cause HCRU categories were comparable between cohorts (**Supplementary Figure S1B**). Mean all-cause total costs were higher for the MMN cohort compared with the MMN-mimic cohort during the preindex and postindex periods (preindex $58 974 vs $48 132 [*P* = .100] and postindex $74 187 vs $50 652 [*P* = .002]) (**Figure 3A, Figure 4B**).

### MMN-Related HCRU and Costs in MMN and MMN-Mimic Cohorts During the Preindex and Postindex Periods

During the preindex period, the MMN cohort (vs MMN-mimic cohort) had significantly higher proportions of patients with an MMN-related ambulatory visit (41% vs 29%, respectively; *P* < .001) and pharmacy use (9% vs 5%, respectively; *P* = .025); the proportion of patients with inpatient stays was significantly lower (6% vs 14%, respectively; *P*<.001) (**Supplementary Figure S2A**). In the postindex period, the MMN cohort (vs MMN-mimic cohort) had a significantly higher proportion of patients with MMN-related ambulatory visits (94% vs 80%, respectively; *P* < .001) and pharmacy use (12% vs 7%, respectively; *P* = .003) (**Supplementary Figure S2B**). For the MMN cohort, the number of MMN-related ambulatory visits was higher during the 12-month postindex period (mean, 7.7 visits) compared with the preindex period (mean, 1.9 visits). A significantly higher proportion of patients in the MMN cohort had MMN-related neurology clinic visits than the MMN-mimic cohort during both preindex and postindex periods (preindex: 26.8% vs 19.5%, respectively; *P* < .011 and postindex: 43.8% vs 28.2%, respectively; *P* < .001). MMN-related healthcare costs were higher for the MMN cohort compared with the MMN-mimic cohort (mean total costs, preindex $23 625 vs $12 890 [*P* = .011] and postindex $39 521 vs $11 938 [*P* < .001]) (**Figure 4A, B**).

## DISCUSSION

In this retrospective study of 904 commercial and Medicare Advantage-insured patients from the Optum Research Database, most patients with MMN received at least 1 alternative diagnosis to MMN. This aligns with a previously published natural history study of patients with MMN in which only 6 of 46 patients were initially diagnosed correctly, with all other patients initially diagnosed with a different condition.^5^ In this study of patients from the Optum Research Database, various types of neuropathies or polyneuropathy (eg, polyneuropathy unspecified, hereditary, and idiopathic neuropathy) were among the most common differential diagnoses observed. The overall observation that differential diagnoses were more common in patients with MMN than in the MMN-mimic cohort is consistent with a higher degree of misdiagnosis for this patient group. This finding is perhaps not surprising given the rarity of MMN relative to many of the MMN-mimic conditions, as well as the complexity of achieving an accurate MMN diagnosis. Significantly more patients with MMN (vs MMN-mimic cohort) received MMN-related medications (including IVIG) and corticosteroids at preindex. More patients with MMN used IVIG at postindex compared with the preindex period. As IVIG is the only approved treatment option and standard of care for patients with MMN, these findings show that appropriate treatment is initiated in only some patients once they are diagnosed (>70% of patients with MMN did not receive IVIG within 12 months postindex), potentially affecting outcomes.^2,6^ Actual MMN-related healthcare costs are likely underestimated given the observed undertreatment with IVIG in this sample.

Our findings suggest that MMN may be frequently misdiagnosed, or the ICD code misattributed. Among the broader group of patients with at least one claim associated with MMN during the identification period, only 37% (n = 336) met the stricter study criteria used to define MMN (of ≥2 nondiagnostic claims associated with MMN). This finding may be due to physicians potentially misapplying or mislabeling patients with other conditions or having a high suspicion of MMN initially that was not supported by subsequent testing.

These findings add to the limited evidence for treatment patterns in patients with MMN and suggest that this gap in appropriate treatment may be associated with a lack of awareness and access to proper treatment, and potentially uncertainty in the diagnosis of MMN.

There are several possible factors that contributed to the reported number of MMN diagnoses and low proportion of patients with MMN being treated with IVIG. Owing to the dispersed nature of centers of excellence for MMN and the decentralized and non-single-payer system in the United States, there may be a proportion of patients with MMN who remain undiagnosed or have had diagnosis codes for the disease misattributed. The interpretation of treatment data may be complicated because of variations in MMN expertise between treating physicians. Physicians with limited experience and understanding of how to treat patients with MMN may inappropriately prescribe medications such as corticosteroids, which can potentially worsen the condition for these patients.^2^ To ensure that proper care is being provided, patients with MMN need to be identified in a timely manner and referred to a neuromuscular specialist.^11^ Further, results showed that 336 patients met the study definition of MMN, but when limiting the definition of the MMN cohort to additionally require that patients be treated with IVIG up to 12 months postindex, only 94 patients were identified.

Although some aspects of HCRU were comparable between patients with MMN and those with MMN-mimic conditions, all-cause and MMN-related costs were higher during preindex and postindex periods for patients with MMN. During both time periods, the difference in costs appears to be driven by pharmacy use and ambulatory visits as opposed to acute care needs such as emergency room visits or inpatient stays as shown in **Supplementary Figure S1and Supplementary Figure S2**. Given that MMN is a chronic and disabling illness that requires continued long-term treatment for disease control, somewhat higher pharmacy and medical costs would be expected. Thus, the increased costs for MMN relative to MMN-mimic conditions may be attributable to disease complexity and the burden associated with the long-term treatment of MMN. For both cohorts, total costs were higher during the postindex period when compared with the preindex period. Given that the cohorts in this study were not balanced, including preindex data allowed for an assessment that encompasses a prolonged view of the patient journey. To our knowledge, this is the first study to report healthcare cost data in US patients with MMN.

CIDP is another type of neuropathy that has a specific variant, multifocal CIDP, which presents similarly to MMN in that it also manifests as asymmetrical weakness.^11^ The economic outcomes reported here are similar to those from previous studies that assessed the economic burden of CIDP in the United States. In a matched case-control study of 790 patients with newly diagnosed CIDP, cases had significantly higher mean total costs (~$116 000 vs $16 000) than a matched control group without CIDP over 2 years of follow-up.^12^ In a stratified exploratory analysis of these findings, patients treated with IVIG monotherapy incurred an average cost of $165 000 for CIDP therapy.^12^ Another study of 73 patients with CIDP reported that all-cause health-plan-paid costs were ~$57 000 annually, with pharmacy costs accounting for more than half (57%) of the total. IVIG treatment was the predominant cost driver for 90% of the pharmacy costs.^13^ Thus, both MMN and CIPD are associated with an increase in total healthcare costs relative to comparator cohorts, which may be driven by IVIG treatment.

Our study had limitations that are common to retrospective observational studies. First, inherent in the use of a claims-based data source, this study involved the selection of patients with access to providers who were aware of MMN, with 43.8% of patients with MMN and 28.2% of patients with MMN-mimic conditions having neurology clinic visits during the postindex period. Therefore, the study did not include those who were not engaged with the healthcare system or did not have access to such providers (ie, no appearance of MMN diagnosis code). Second, before the release of the ICD-10-CM code for MMN on October 1, 2016, and during the early coding-adoption phase, patients with MMN may have been classified under other ICD-9-CM or ICD-10-CM codes, especially CIDP. Although the identification period was defined to start on October 1, 2016, it was limited by provider familiarity with the diagnosis code and confirmation of the diagnosis. This may have led to patients with MMN continuing to carry a diagnosis code for another condition and being excluded from the MMN cohort or from the entire study population. In addition, patients with MMN in our study were categorized by index year; thus, we were unable to identify the number of patients who had confirmed cases of MMN by year. Certain clinical variables (eg, anti-GM1 test results) were also not directly available within this claims database. In the absence of these clinical covariates, the study findings were subject to unobserved confounding factors, which are typical in observational studies. Lastly, a misclassification bias may have been present when classifying MMN and MMN-mimic patients, given the limitations previously discussed. Cohort definitions were based on clinical and HCRU characteristics and supported by literature and expert input; validation of cohort definitions may be explored in future work (eg, comparison with another data source) to assess the extent of this bias. Nonetheless, we believe this database provided high longitudinal integrity, patient retention, and a detailed overview of real-world HCRU and clinical outcomes for a large subset of the US population with MMN.

In summary, this study used claims data to address the evidence gap regarding the characteristics, treatment patterns, and associated healthcare burden of patients with MMN and MMN-mimic conditions. The findings of this study further highlight the need for physician education and improvements in the diagnosis of MMN to distinguish the disease from similar conditions, decrease the time to diagnosis, and ensure patient access to appropriate treatment. Facilitating accurate diagnoses, in turn, may lead to an increase in the treatment of MMN with IVIG. Future research should explore how the costs of treating MMN may be associated with indirect costs, clinical outcomes, patient quality of life, and functional outcomes.

### Ethics Approval and Patient Consent

Not applicable. This retrospective analysis of de-identified administrative claims is exempt from human subjects research review.

## Supplementary Material

Online Supplementary Material

## Data Availability

Aggregated/summary data that support the findings of this study are available from the corresponding author upon reasonable request.

## References

[1] Hameed S., Cascella M. (2022). StatPearls.

[2] Joint Task Force of the EFNS and the PNS (2010). European Federation of Neurological Societies/Peripheral Nerve Society guideline on management of multifocal motor neuropathy. Report of a joint task force of the European Federation of Neurological Societies and the Peripheral Nerve Society--first revision. J Peripher Nerv Syst.

[3] Cats E. A., van der Pol W. L., Piepers S.. (2010). Correlates of outcome and response to IVIg in 88 patients with multifocal motor neuropathy. Neurology.

[4] Lawson V., Robbins N. (2018). The potential misdiagnosis of multifocal motor neuropathy as amyotrophic lateral sclerosis—a case series. US Neurol.

[5] Taylor B. V., Wright R. A., Harper C. M., Dyck P. J. (2000). Natural history of 46 patients with multifocal motor neuropathy with conduction block. Muscle Nerve.

[6] GBS/CIDP Foundation International (2021). MULTIFOCAL MOTOR NEUROPATHY (MMN).

[7] GBS/CIDP Foundation International (2021). Centers of excellence.

[8] Olney R. K., Lewis R. A., Putnam T. D., Campellone J. V., Jr., American Association of Electrodiagnostic M. (2003). Consensus criteria for the diagnosis of multifocal motor neuropathy. Muscle Nerve.

[9] Dimachkie M. M., Barohn R. J., Katz J. (2013). Multifocal motor neuropathy, multifocal acquired demyelinating sensory and motor neuropathy, and other chronic acquired demyelinating polyneuropathy variants. Neurol Clin.

[10] SAS Institute Inc. (2023). SAS/STAT® 15.3 User’s Guide.

[11] Allen J. A., Clarke A. E., Harbo T. (2024). A practical guide to identify patients with multifocal motor neuropathy, a treatable immune-mediated neuropathy. Mayo Clin Proc Innov Qual Outcomes.

[12] Divino V., Mallick R., DeKoven M., Krishnarajah G. (2018). The economic burden of CIDP in the United States: a case-control study. PLoS One.

[13] Guptill J. T., Bromberg M. B., Zhu L.. (2014). Patient demographics and health plan paid costs in chronic inflammatory demyelinating polyneuropathy. Muscle Nerve.

