# Supplementary material for: Characteristics, Treatment Patterns, Healthcare Resource Utilization, and Costs Among Patients with Multifocal Motor Neuropathy: A US Claims Database Cohort Study
Source: J Health Econ Outcomes Res. 2025 Jun 26;12(1):261–8. doi: 10.36469/001c.140817 (PMC12205905; doi:10.36469/001c.140817)
Supplement: Online Supplementary Material [file jheor_2025_12_1_140817_291037.pdf]

Journal of Health Economics and Outcomes Research

Online Supplementary Material

Characteristics, Treatment Patterns, Healthcare Resource Utilization, and Costs Among Patients with Multifocal Motor Neuropathy: A US Claims Database Cohort Study. *JHEOR*. 2025;12(1):261-268.
doi:10.36469/jheor.2025.140817

Diagnostic Procedures and Medications

Figure S1: All-Cause HCRU in the Study Population During the (A) Preindex and (B) Postindex Period

Figure S2: MMN-Related HCRU in the Study Population During the (A) Preindex and (B) Postindex Period

Table S1: ICD-10-CM Codes Associated With MMN-Mimic Conditions

Table S2: Specialty of the Healthcare Provider Submitting Claims For Diagnostic Tests Associated With an MMN Diagnosis During the Preindex and Postindex Periods

This supplementary material has been provided by the authors to give readers additional information about their work.

This is an open-access article distributed under the terms of the Creative Commons Attribution 4.0 International License (CCBY-4.0). View this license's legal deed at http://creativecommons.org/licenses/by/4.0 and legal code at http://creativecommons.org/licenses/by/4.0/legalcode for more information.

DIAGNOSTIC PROCEDURES AND MEDICATIONS

MMN-Related Diagnostic Procedures and Medications

Diagnostic assessments were measured in the preindex period and 3-month diagnostic follow-up period (ie, the diagnostic window surrounding the index date). Variables were created separately to reflect timing of utilization (preindex vs diagnostic follow-up period).

- **MMN-related diagnostic procedures:** Indicators and counts were created for patients undergoing various diagnostic procedures using procedure codes on medical claims (including diagnostic claims). Counts were defined as the count of unique dates of service with ≥1 procedure code of interest. Procedures and assessments included:

  - **Electrophysiological analysis:** indicators and counts on different dates were identified for patients with ≥1 medical claim for a nerve conduction study (NCS), electromyography (EMG), or nerve ultrasound (defined as a nonspecific/general location ultrasound procedure code on the same medical claim with an ICD-10-CM diagnosis code for upper motor neuron disorder, MMN, MMN-mimic conditions, or defined neuromuscular sign/symptom) using procedure codes during the preindex or follow-up period.

  - **Magnetic resonance imaging (MRI)**: indicators and counts on different dates were identified for patients with ≥1 medical claim for an MRI using procedure codes during the preindex or follow-up period. Separate indicators categorized the location of the MRI.

    - **MRI of the spine/brachial plexus:** Spinal MRI was noted as a rule-out for Hirayama disease and brachial plexus swelling a supporting criterion for MMN diagnosis.
    - **Other MRI:** MRI of other neurological-related upper extremities (eg, head/brain, spine area, joint, extremities) or nonspecific as to location.

  - **Lumbar puncture/cerebral spinal fluid (CSF) collection**: Indicators and counts on different dates were identified for patients with ≥1 medical claim for a lumbar puncture or CSF collection using procedure codes during the preindex or follow-up period.

  - **MMN-associated antibody tests:** Indicators and counts on different dates were identified for patients with ≥1 medical claim during the preindex or follow-up periods. Tests were defined as MMN-associated if a procedure code for antibody testing (general codes) presented on the same medical claim with an ICD-10-CM diagnosis code in the primary position for upper motor neuron disorder, MMN, MMN-mimic conditions, or defined neuromuscular signs/symptoms.

Specified medications were measured in the preindex period and follow-up period.

- **MMN-related medications:** Indicators and counts (ie, unique dates of service) were created for patients receiving treatments used on or off-label for MMN and related conditions using both medical and pharmacy claims during the preindex period and follow-up period.

The following were categorized:

  - **Immunoglobulins (IVIG or SCIG):** Indicator variables for overall use and use of specific formulations (intravenous [IV] or subcutaneous [SC]) were created to identify immunoglobulin (IG) use using medical and pharmacy claims. Variables were created separately to reflect timing of utilization (preindex period vs follow-up period). Additionally, patterns of immunoglobulins were assessed

Note: SCIG was not currently approved for MMN use and infrequent utilization was expected.

    - Indicators of use by brand name were created. If the brand name was not available (ie, generic HCPCS code), then the brand was listed as unknown. If multiple brand products were used during the time period, then this was noted as use for each individual brand product.
    - Indicators of medical vs pharmacy claims were created.
    - IVIG vs SCIG products were also categorized. Products that could be administered as both IV and SC were grouped under IVIG.

- **Immunologics/immunosuppressives:** Indicator and count variables for overall use and use of specific classes was created to identify immunologic/immunosuppressive medications using pharmacy and medical claims (Note: these agents were indicated for other immunologic conditions and potentially could be used off-label for MMN). Variables were created separately to reflect timing of utilization (preindex period vs follow-up period).

    - **Rituximab (including biosimilars):** Identified using ≥1 medical and pharmacy claims for rituximab or rituximab biosimilars
    - **Cyclophosphamide**: Identified using ≥1 medical and pharmacy claims for cyclophosphamide
    - **Eculizumab**: Identified using ≥1 medical and pharmacy claims for eculizumab
    - **Other immunomodulators/immunosuppressives**: Azathioprine, cyclosporine, interferon beta-1a, methotrexate sodium, and mycophenolate mofetil

  - **Any disease-modifying treatment**: An overall indicator was created to identify treatment received with either immunoglobulin or immunologic/immunosuppressives. Variables were created separately to reflect timing of utilization (preindex period vs follow-up period).

MMN-Mimic Differential Diagnoses and Treatments

The following reflect differential diagnoses, nonspecific neuromuscular signs or symptoms, or diagnostic exclusions for MMN as well as treatments that were ineffective in MMN or were indicated for other differential diagnoses. These were measured during the preindex and follow-up periods.

- **MMN-mimic differential diagnoses:** Indicator variables and counts (ie, unique dates of service) were created to identify patients with ≥1 of the following commonly misdiagnosed conditions as well as by condition using ICD-10-CM codes in any position on medical claims (including diagnostic claims). These conditions were identified among all diagnosis codes documented in the 4-year preindex period prior to the index MMN diagnosis date and based on differential diagnoses associated with MMN. Variables were created separately to reflect timing of utilization (preindex period vs follow-up period).
    - Amyotrophic lateral sclerosis
    - Upper extremity neuropathies
        - Carpal tunnel syndrome
        - Brachial plexus disorders
        - Cervical root disorders
        - Median, ulnar, and radial nerve disorders/lesions
        - Upper limb causalgia/mononeuropathy
    - Chronic inflammatory demyelinating polyneuritis
    - Guillain-Barre syndrome
    - Hereditary and idiopathic neuropathies
        - Hereditary and idiopathic neuropathy unspecified
        - Other hereditary and idiopathic neuropathies
        - Hereditary motor and sensory neuropathy
        - Neuropathy (general)
    - Progressive muscular atrophy
    - Inflammatory polyneuropathy unspecified
    - Polyneuropathy unspecified
    - Lumbosacral disorders
        - Lumbosacral plexus disorders
        - Lumbosacral root disorders
    - Diabetes mellitus (type 1, type 2, or other) with diabetic neuropathy
        - Diabetic neuropathy unspecified
        - Diabetic polyneuropathy
        - Other diabetic neuropathy

    The following conditions were added following review of the diagnosis codes identified:

    - Porphyria
    - Drug-related/substance-related neurotoxicities
        - Lead
        - Drugs and other (eg, radiation, alcohol, other toxin)
    - Sarcoidosis
        - Sarcoid neuropathy or unspecified
        - Sarcoidosis - other site
    - Hirayama disease (nonspecific codes for spinal or other primary muscular disorders)
    - Radiculopathy
        - Unlisted cause
        - Specified cause
    - Other neuropathy (eg, secondary to other causes such as infection, rheumatoid arthritis, or unspecified)
    - Nerve injury

  - Motor neuron disease unspecified
  - Spinal stenosis
  - Monoplegia
- **Ill-defined neuromuscular sign/symptom**: An indicator was created for patients with ≥1 medical claims containing ICD-10-CM codes for symptoms and signs involving the nervous and musculoskeletal systems (R25-R29, excluding ocular torticollis and loss of height) in any position (eg, abnormal gait, ataxia). Variables were created separately to reflect timing of utilization (preindex period vs follow-up period).
- **Exclusion criteria for MMN diagnosis:** An overall indicator and specific criterion indicators were created for patients with ≥1 medical claims containing ICD-10-CM codes for a possible exclusion criterion (differential diagnoses) for MMN in any position. Variables were created separately to reflect timing of utilization (preindex period vs follow-up period).
  - Sensory symptoms (eg, paresthesia)
  - Bulbar involvement (eg, dysphagia, general). Excludes dysphagia from known causes (ie, post-stroke dysphagia)
  - Upper motor neuron disease (eg, spinal lesions)
  - Upper extremity symmetric weakness (eg, bilateral weakness)
- **MMN-mimic related procedures**: Indicators and counts (different dates of service) were created for patients undergoing various procedures that were not effective for MMN or used in other neuromuscular conditions using CPT codes on medical claims. Variables were created separately to reflect timing of utilization (preindex period vs follow-up period).
  - **Plasmapheresis procedures**: An indicator variable and count were identified for patients with ≥1 medical claim with a CPT code for plasmapheresis. Patients with MMN usually did not respond to or may have worsened with this treatment.
- **MMN-mimic related medications**: Indicators and counts (different dates of service) were created for patients using both medical and pharmacy claims to identify medications that were either not effective for MMN or indicated for other neurological diseases commonly diagnosed in patients with MMN. Variables were created separately to reflect timing of utilization (preindex period vs follow-up period).
  - **Systemic corticosteroids**: An indicator variable was created to identify any steroid use using pharmacy and medical claims. Additionally, categorical variables were created based on total days' supply summed across all claims: 0 to 14 days, 15 to 30 days, 31 to 60 days, and 61+ days. Specifically, systemic corticosteroids were included (eg, methylprednisone). The days' supplies from pharmacy claims and medical claims (assumed 1-day supply per unique medical claim date of service) were totaled. Additionally, a cross frequency of indicators was used to assess patients with systemic corticosteroid use during the preindex period as well as during the follow-up period.

    Note: Although patients received steroid treatment that was indicated for other MMN-mimic conditions (eg, CIDP), patients with MMN usually did not respond to or may have worsened with this treatment. In MMN, steroids may have been used in severe disease or in patients with axonal loss (per anecdotal comment from providers).
  - **ALS medications:** ≥1 medical or pharmacy claims for FDA-approved ALS medications: riluzole or edaravone.
  - **Neuropathic pain medications**: Indicator and count variables were created using pharmacy claims for gabapentin or pregabalin.

    Note: These medications may have indicated sensory dysfunction (eg, paresthesia), an exclusion criterion for MMN diagnosis, but also had indications for epilepsy.
- **Other pain medications:** Indicator and count variables were created using pharmacy claims for other narcotics and analgesic prescription medication (eg, opioids, non-steroidal anti-inflammatory drugs). Other pain medications were summarized overall and by opioid or opioid-like narcotic (eg, oxycodone, tramadol) vs nonopioid pain medications.

Figure S1. All-Cause HCRU in the Study Population During the (**A**) Preindex and (**B**) Postindex Period

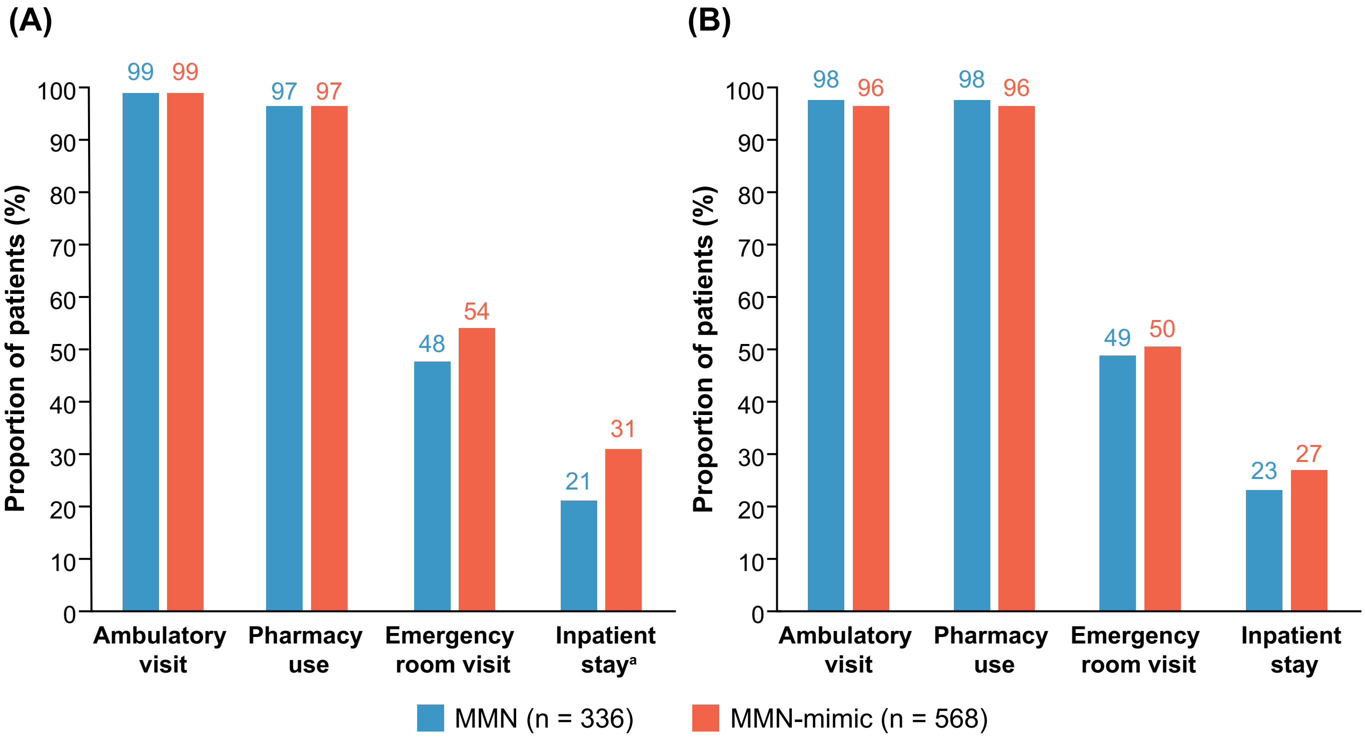

Abbreviations: HCRU, healthcare resource utilization; MMN, multifocal motor neuropathy.
[a]Statistically significant ($P<.05$) difference between MMN and MMN-mimic cohorts.

Figure S2. MMN-Related HCRU in the Study Population During the (**A**) Preindex and (**B**) Postindex Period

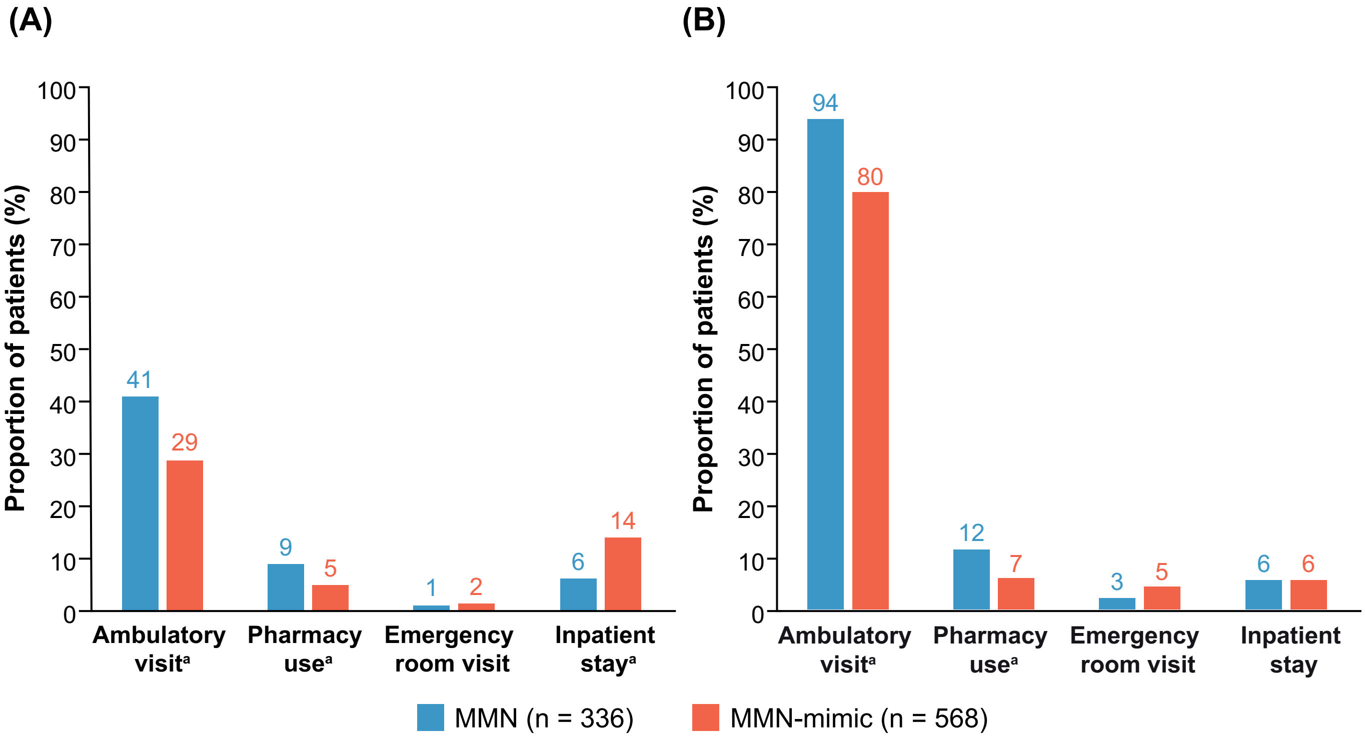

Abbreviations: HCRU, healthcare resource utilization; MMN, multifocal motor neuropathy.
Note: HCRU was defined as MMN-related if claims had an MMN diagnosis code or had a code for a medication/diagnostic test for MMN.
[a]Statistically significant ($P<.05$) difference between MMN and MMN-mimic cohorts

Table S1. ICD-10-CM Codes Associated With MMN-Mimic Conditions

ICD-10-CM Code	Description
G540	Brachial plexus disorders
G542	Cervical root disorders, not elsewhere classified
G5600	Carpal tunnel syndrome, unspecified upper limb
G5601	Carpal tunnel syndrome, right upper limb
G5602	Carpal tunnel syndrome, left upper limb
G5603	Carpal tunnel syndrome, bilateral upper limbs
G5610	Other lesions of median nerve, unspecified upper limb
G5611	Other lesions of median nerve, right upper limb
G5612	Other lesions of median nerve, left upper limb
G5613	Other lesions of median nerve, bilateral upper limbs
G5620	Lesion of ulnar nerve, unspecified upper limb
G5621	Lesion of ulnar nerve, right upper limb
G5622	Lesion of ulnar nerve, left upper limb
G5623	Lesion of ulnar nerve, bilateral upper limbs
G5630	Lesion of radial nerve, unspecified upper limb
G5631	Lesion of radial nerve, right upper limb
G5632	Lesion of radial nerve, left upper limb
G5633	Lesion of radial nerve, bilateral upper limbs
G5640	Causalgia of unspecified upper limb
G5641	Causalgia of right upper limb
G5642	Causalgia of left upper limb
G5643	Causalgia of bilateral upper limbs
G5680	Other specified mononeuropathies of unspecified upper limb
G5681	Other specified mononeuropathies of right upper limb
G5682	Other specified mononeuropathies of left upper limb
G5683	Other specified mononeuropathies of bilateral upper limbs
G5690	Unspecified mononeuropathy of unspecified upper limb
G5691	Unspecified mononeuropathy of right upper limb
G5692	Unspecified mononeuropathy of left upper limb
G5693	Unspecified mononeuropathy of bilateral upper limbs
E0840	Diabetes mellitus due to underlying condition with diabetic neuropathy, unspecified
E0842	Diabetes mellitus due to underlying condition with diabetic polyneuropathy
E0940	Drug or chemical induced diabetes mellitus with neurological complications with diabetic neuropathy, unspecified
E0942	Drug or chemical induced diabetes mellitus with neurological complications with diabetic polyneuropathy
E1040	Type 1 diabetes mellitus with diabetic neuropathy, unspecified
E1042	Type 1 diabetes mellitus with diabetic polyneuropathy
E1140	Type 2 diabetes mellitus with diabetic neuropathy, unspecified
E1340	Other specified diabetes mellitus with diabetic neuropathy, unspecified
E1342	Other specified diabetes mellitus with diabetic polyneuropathy
G1221	Amyotrophic lateral sclerosis
G1225	Progressive spinal muscle atrophy
G541	Lumbosacral plexus disorders
G544	Lumbosacral root disorders, not elsewhere classified
G545	Neuralgic amyotrophy
G546	Phantom limb syndrome with pain
G547	Phantom limb syndrome without pain

Table S1. ICD-10-CM Codes Associated With MMN-Mimic Conditions

ICD-10-CM Code	Description
G548	Other nerve root and plexus disorders
G549	Nerve root and plexus disorder, unspecified
G55	Nerve root and plexus compressions in diseases classified elsewhere
G603	Idiopathic progressive neuropathy
G608	Other hereditary and idiopathic neuropathies
G609	Hereditary and idiopathic neuropathy, unspecified
G610	Guillain-Barre syndrome
G6181	Chronic inflammatory demyelinating polyneuritis
G6189	Other inflammatory polyneuropathies
G619	Inflammatory polyneuropathy, unspecified
G629	Polyneuropathy, unspecified
G63	Polyneuropathy in diseases classified elsewhere
E800	Hereditary erythropoietic porphyria
E801	Porphyria cutanea tarda
E8020	Unspecified porphyria
E8021	Acute intermittent (hepatic) porphyria
E8029	Other porphyria
G620	Drug-induced polyneuropathy
G621	Alcoholic polyneuropathy
R7871	Abnormal lead level in blood
T560X1A	Toxic effect of lead and its compounds, accidental (unintentional), initial encounter
T560X1D	Toxic effect of lead and its compounds, accidental (unintentional), subsequent encounter
T560X1S	Toxic effect of lead and its compounds, accidental (unintentional), sequela
T560X2A	Toxic effect of lead and its compounds, intentional self-harm, initial encounter
T560X2D	Toxic effect of lead and its compounds, intentional self-harm, subsequent encounter
T560X2S	Toxic effect of lead and its compounds, intentional self-harm, sequela
T560X3A	Toxic effect of lead and its compounds, assault, initial encounter
T560X3D	Toxic effect of lead and its compounds, assault, subsequent encounter
T560X3S	Toxic effect of lead and its compounds, assault, sequela
T560X4A	Toxic effect of lead and its compounds, undetermined, initial encounter
T560X4D	Toxic effect of lead and its compounds, undetermined, subsequent encounter
T560X4S	Toxic effect of lead and its compounds, undetermined, sequela
Z77011	Contact with and (suspected) exposure to lead
D8682	Multiple cranial nerve palsies in sarcoidosis
D8689	Sarcoidosis of other sites
D869	Sarcoidosis, unspecified
D860	Sarcoidosis of lung
D861	Sarcoidosis of lymph nodes
D862	Sarcoidosis of lung with sarcoidosis of lymph nodes
D863	Sarcoidosis of skin
D8681	Sarcoid meningitis
D8683	Sarcoid iridocyclitis
D8684	Sarcoid pyelonephritis
D8685	Sarcoid myocarditis
D8686	Sarcoid arthropathy
D8687	Sarcoid myositis

Table S1. ICD-10-CM Codes Associated With MMN-Mimic Conditions

ICD-10-CM Code	Description
G128	Other spinal muscular atrophies and related syndromes
G718	Other primary disorders of muscles
G719	Primary disorder of muscle, unspecified
G580	Intercostal neuropathy
M4720	Other spondylosis with radiculopathy, site unspecified
M4721	Other spondylosis with radiculopathy, occipito-atlanto-axial region
M4722	Other spondylosis with radiculopathy, cervical region
M4723	Other spondylosis with radiculopathy, cervicothoracic region
M4724	Other spondylosis with radiculopathy, thoracic region
M4725	Other spondylosis with radiculopathy, thoracolumbar region
M4726	Other spondylosis with radiculopathy, lumbar region
M4727	Other spondylosis with radiculopathy, lumbosacral region
M4728	Other spondylosis with radiculopathy, sacral and sacrococcygeal region
M47811	Spondylosis without myelopathy or radiculopathy, occipito-atlanto-axial region
M47812	Spondylosis without myelopathy or radiculopathy, cervical region
M47813	Spondylosis without myelopathy or radiculopathy, cervicothoracic region
M47814	Spondylosis without myelopathy or radiculopathy, thoracic region
M47815	Spondylosis without myelopathy or radiculopathy, thoracolumbar region
M47816	Spondylosis without myelopathy or radiculopathy, lumbar region
M47817	Spondylosis without myelopathy or radiculopathy, lumbosacral region
M47818	Spondylosis without myelopathy or radiculopathy, sacral and sacrococcygeal region
M47819	Spondylosis without myelopathy or radiculopathy, site unspecified
M48062	Spinal stenosis, lumbar region with neurogenic claudication
M5010	Cervical disc disorder with radiculopathy, unspecified cervical region
M5011	Cervical disc disorder with radiculopathy, high cervical region
M5012	Cervical disc disorder with radiculopathy, mid-cervical region
M50121	Cervical disc disorder at C4-C5 level with radiculopathy
M50122	Cervical disc disorder at C5-C6 level with radiculopathy
M50123	Cervical disc disorder at C6-C7 level with radiculopathy
M5013	Cervical disc disorder with radiculopathy, cervicothoracic region
M5114	Intervertebral disc disorders with radiculopathy, thoracic region
M5115	Intervertebral disc disorders with radiculopathy, thoracolumbar region
M5116	Intervertebral disc disorders with radiculopathy, lumbar region
M5117	Intervertebral disc disorders with radiculopathy, lumbosacral region
M5410	Radiculopathy, site unspecified
M5411	Radiculopathy, occipito-atlanto-axial region
M5412	Radiculopathy, cervical region
M5413	Radiculopathy, cervicothoracic region
M5414	Radiculopathy, thoracic region
M5415	Radiculopathy, thoracolumbar region
M5416	Radiculopathy, lumbar region
M5417	Radiculopathy, lumbosacral region
M5418	Radiculopathy, sacral and sacrococcygeal region
M5430	Sciatica, unspecified side
M5431	Sciatica, right side
M5432	Sciatica, left side

Table S1. ICD-10-CM Codes Associated With MMN-Mimic Conditions

ICD-10-CM Code	Description
M5440	Lumbago with sciatica, unspecified side
M5441	Lumbago with sciatica, right side
M5442	Lumbago with sciatica, left side
M542	Cervicalgia
A5043	Late congenital syphilitic polyneuropathy
A5215	Late syphilitic neuropathy
B0223	Postherpetic polyneuropathy
B2684	Mumps polyneuropathy
B2701	Gammaherpesviral mononucleosis with polyneuropathy
B2711	Cytomegaloviral mononucleosis with polyneuropathy
B2781	Other infectious mononucleosis with polyneuropathy
B2791	Infectious mononucleosis, unspecified with polyneuropathy
E0841	Diabetes mellitus due to underlying condition with diabetic mononeuropathy
E0843	Diabetes mellitus due to underlying condition with diabetic autonomic (poly)neuropathy
E0941	Drug or chemical induced diabetes mellitus with neurological complications with diabetic mononeuropathy
E0943	Drug or chemical induced diabetes mellitus with neurological complications with diabetic autonomic (poly)neuropathy
E1041	Type 1 diabetes mellitus with diabetic mononeuropathy
E1043	Type 1 diabetes mellitus with diabetic autonomic (poly)neuropathy
E1141	Type 2 diabetes mellitus with diabetic mononeuropathy
E1142	Type 2 diabetes mellitus with diabetic polyneuropathy
E1143	Type 2 diabetes mellitus with diabetic autonomic (poly)neuropathy
E1341	Other specified diabetes mellitus with diabetic mononeuropathy
E1343	Other specified diabetes mellitus with diabetic autonomic (poly)neuropathy
E71522	Adrenomyeloneuropathy
G130	Paraneoplastic neuromyopathy and neuropathy
G5790	Unspecified mononeuropathy of unspecified lower limb
G5791	Unspecified mononeuropathy of right lower limb
G5792	Unspecified mononeuropathy of left lower limb
G5793	Unspecified mononeuropathy of bilateral lower limbs
G587	Mononeuritis multiplex
G589	Mononeuropathy, unspecified
G59	Mononeuropathy in diseases classified elsewhere
G600	Hereditary motor and sensory neuropathy
G602	Neuropathy in association with hereditary ataxia
G611	Serum neuropathy
G622	Polyneuropathy due to other toxic agents
G6281	Critical illness polyneuropathy
G6282	Radiation-induced polyneuropathy
G651	Sequelae of other inflammatory polyneuropathy
G652	Sequelae of toxic polyneuropathy
G9009	Other idiopathic peripheral autonomic neuropathy
G990	Autonomic neuropathy in diseases classified elsewhere
H462	Nutritional optic neuropathy
M0550	Rheumatoid polyneuropathy with rheumatoid arthritis of unspecified site
M05511	Rheumatoid polyneuropathy with rheumatoid arthritis of right shoulder

Table S1. ICD-10-CM Codes Associated With MMN-Mimic Conditions

ICD-10-CM Code	Description
M05512	Rheumatoid polyneuropathy with rheumatoid arthritis of left shoulder
M05519	Rheumatoid polyneuropathy with rheumatoid arthritis of unspecified shoulder
M05521	Rheumatoid polyneuropathy with rheumatoid arthritis of right elbow
M05522	Rheumatoid polyneuropathy with rheumatoid arthritis of left elbow
M05529	Rheumatoid polyneuropathy with rheumatoid arthritis of unspecified elbow
M05531	Rheumatoid polyneuropathy with rheumatoid arthritis of right wrist
M05532	Rheumatoid polyneuropathy with rheumatoid arthritis of left wrist
M05539	Rheumatoid polyneuropathy with rheumatoid arthritis of unspecified wrist
M05541	Rheumatoid polyneuropathy with rheumatoid arthritis of right hand
M05542	Rheumatoid polyneuropathy with rheumatoid arthritis of left hand
M05549	Rheumatoid polyneuropathy with rheumatoid arthritis of unspecified hand
M05551	Rheumatoid polyneuropathy with rheumatoid arthritis of right hip
M05552	Rheumatoid polyneuropathy with rheumatoid arthritis of left hip
M05559	Rheumatoid polyneuropathy with rheumatoid arthritis of unspecified hip
M05561	Rheumatoid polyneuropathy with rheumatoid arthritis of right knee
M05562	Rheumatoid polyneuropathy with rheumatoid arthritis of left knee
M05569	Rheumatoid polyneuropathy with rheumatoid arthritis of unspecified knee
M05571	Rheumatoid polyneuropathy with rheumatoid arthritis of right ankle and foot
M05572	Rheumatoid polyneuropathy with rheumatoid arthritis of left ankle and foot
M05579	Rheumatoid polyneuropathy with rheumatoid arthritis of unspecified ankle and foot
M0559	Rheumatoid polyneuropathy with rheumatoid arthritis of multiple sites
M3483	Systemic sclerosis with polyneuropathy

Abbreviations: ICD-10-CM, *International Classification of Diseases, Tenth Revision, Clinical Modification*; MMN, multifocal motor neuropathy.

Table S2. Specialty of the Healthcare Provider Submitting Claims for Diagnostic Tests Associated With an MMN Diagnosis During the Preindex and Postindex Periods

Healthcare Specialty, n (%)	Overall Study Population (N = 904)	MMN (n = 336)	MMN-Mimic (n = 568)	MMN vs MMN-Mimic *P* Value
Preindex period				
Neurology	185 (20.5)	80 (23.8)	105 (18.5)	0.055
Occupational/physical/rehabilitation medicine	32 (3.5)	12 (3.6)	20 (3.5)	0.968
Primary care[a,c]	<9 (<1.0)	<5 (<1.5)	<5 (<0.9)	0.272
Orthopedic surgery[c]	<9 (<1.0)	<5 (<1.5)	<5 (<0.9)	0.614
Postindex period				
Neurology	199 (22.0)	86 (25.6)	113 (19.9)	0.046[b]
Primary care[a]	96 (10.6)	40 (11.9)	56 (9.9)	0.335
Occupational/physical/rehabilitation medicine	28 (3.1)	9 (2.7)	19 (3.4)	0.576
Orthopedic surgery[c]	<12 (<1.3)	<5 (<1.5)	7 (1.2)	0.351
Physical/occupational therapy services[c]	<9 (<1.0)	<5 (<1.5)	<5 (<0.9)	0.117

Abbreviation: MMN, multifocal motor neuropathy.
[a]Family or general practice, internal medicine, pediatrics, obstetrics/gynecology or geriatric provider.
[b]Statistically significant ($P < .05$) difference.
[c]Data are masked where patient numbers are <5.
